# Editorial: Quality of stroke care: what could be improved, and how?

**DOI:** 10.3389/fneur.2023.1250872

**Published:** 2023-07-11

**Authors:** Aleksandras Vilionskis, Janika Korv

**Affiliations:** ^1^Clinic of Neurology and Neurosurgery, Vilnius University, Vilnius, Lithuania; ^2^Department of Neurology and Neurosurgery, University of Tartu, Tartu, Estonia

**Keywords:** quality, stroke care, stroke therapy, stroke unit, stroke rehabilitation

Stroke is one of the leading contributors to long-term disability and mortality worldwide (1). Its direct and undirect burden is substantial and perhaps will rise in future due to the aging of the population (2). Reperfusion treatment allows to decrease the disability among the survivors, but only a small amount of acute stroke patients receive that modern therapy (3, 4). There are several international initiatives aiming at improvement of care of acute stroke patients and reducing their mortality as disability as well. The stroke care pathway includes all stages from prehospital until rehabilitation and post-stroke care. There are many challenges in each step, and it is crucial to identify the gaps on each stage and to find the ways to improve stroke care.

The aim of our special Research Topic “*Quality of stroke care: what could be improved and how?*” was designed to present the real-world situation regarding stroke services at all stages across different countries and provide some practical recommendations to improve acute stroke care in daily practice. The Research Topic includes 15 manuscripts: 1 review, 11 original research, 1 perspective, 1 study protocol, and 1 brief research report. The research has been conducted in different world regions, including Asia, Europe, USA, and the Middle East.

Chen M. et al. performed an original review of bibliometric analysis of stroke and quality of life and showed, that during the last more than two decades the number of publications increased significantly in all parts of the world. Hot topics in stroke research, main research institutions and contributors in this field were identified.

Al Hashmi et al. evaluated the availability of resources for the management of acute stroke in the Middle East, North Africa, and neighboring areas (MENA+). The study revealed significant differences in stroke care between low-income and high-income countries and stressed the importance of development of stroke target programs. Hwong et al. studied the gaps of implementation of intravenous thrombolysis in Malaysia. The analysis showed that delayed presentation at the scene (67.6%) was identified as the primary factor contributing to the low utilization rate of intravenous thrombolysis. The authors note that the facilitative work process and cohesiveness of team members are the important factors for implementation of modern stroke treatments into daily clinical practice. Ganti et al. studied the impact of arrival time to the emergency department on door-to-needle time of thrombolytic treatment. A single hospital data showed that nighttime, lack of dedicated stroke team, longer time-to-CT read and arrival as walk-in are associated with the longer door-to-needle time. These data confirm the results from previous studies (5, 6).

Modern guidelines recommend using a stent-retriever for mechanical thrombectomy for the treatment of acute ischemic stroke due to large vessel occlusion (7). However, many centers use other techniques, including aspiration. Narloch et al. show that the success of aspiration-based first-pass recanalization did not decrease significantly with increasing age and might not be avoided in the elderly patients.

Pneumonia is a common post-stroke complication with the prevalence up to 38% (8). Clinical studies confirm that pneumonia is a preventable condition (9). Studies have shown that dysphagia is an independent risk factor of pneumonia, so early diagnosis and treatment of dysphagia result in the decreasing rate of post-stroke pneumonia and improve the outcome (10). Zhang et al. evaluated the effectiveness of smart health-based rehabilitation on patients with dysphagia in a case-control study of 60 poststroke dysphagia patients. Their findings confirm the significant positive effect of smart rehabilitation compared to routine rehabilitation. In another quasi-intervention pilot study of 120 patients Zheng et al. showed that nurse-led hierarchical management based on acute ischemic stroke – associated pneumonia score is useful tool to reduce the incidence of pneumonia.

Up to 50–60% of acute stroke patients have disability and require rehabilitation and post-stroke long-term care. Sarzyńska-Długosz analysis offers a comprehensive understanding of the ideal long-term care model while shedding light on the real-world obstacles faced in putting that model into practice. Chen M. et al. found in their retrospective study that more than half of acute ischemic stroke patients have risk of malnutrition, and severe neurological deficit on admission and older age are its independent risk factors. These results allow us to identify the target group of patients to whom more attention should be paid. Susts et al. evaluated and confirmed that physical inactivity before stroke is the independent predictor of dependency in basic activities of daily living after stroke. Other predictors are older age, female sex, pre-stroke living conditions, previous stroke, and severity of stroke on admission. Pan et al. found that around 50% of young and middle-aged stroke survivors do not return to work after stroke. Among others, they identified the risk factors such as older age, female sex, low education, and no medical insurance. Another conclusion is that there is a direct relationship between the ability to return to work and health-related quality of life. The authors conclude that specific interventions are needed to facilitate returning to work and improving the quality of life after a stroke. Xie et al. investigated the relation between hemiplegic shoulder pain and subluxation and found that there is a high frequency of shoulder pain and local injury after stroke. The authors provide specific recommendations for rehabilitation for this patients group, which can be quickly implemented into the daily practice. Bae et al. analyzed the utilization of post-stroke rehabilitation prior to the introduction of the post-acute rehabilitation system in South Korea in 2017. The authors conclude that before the nationwide rehabilitation system, the rehabilitation treatment was over- and under-supplied. The study confirms the need for development of post-acute rehabilitation system that specifies the subject, duration, and intensity of rehabilitation treatment.

In contrast to acute ischemic stroke there are only a few recommendations for the treatment of intracerebral hemorrhage (11). The benefit of surgical evacuation of intracerebral hematoma is still an unanswered question. Ratcliff et al. present the protocol for a new multi-centered randomized trial. This trial is addressed to patients with supratentorial spontaneous intracerebral hemorrhage using minimally invasive trans-sulcal parafascicular surgery approach. The results of the trial were presented during the 9th European Stroke Organization Conference in May 2023.

An abdominal aortic aneurysm is a dangerous, potentially fatal, but often underdiagnosed condition (12). There is evidence that the prevalence of abdominal aortic aneurysm is higher among TIA patients (13). Loban et al. presents the results of local screening programs in elderly men over past decade, who have had ischemic stroke or TIA. They suggest that there is a strong basis for recommendation to screen acute stroke patients over 60 years for this condition.

In conclusion, the “*Quality of stroke care: what could be improved and how?*” Research Topic provides the latest data in this field. The 15 studies cover all areas of acute stroke from prehospital stage until rehabilitation and provide new insights into improvement of quality of stroke care. Conduction of studies in different regions results in broad implementation of new knowledge into daily clinical practice. We believe that this Research Topic will be a useful reading for improvement of acute stroke care and mapping future directions for research.

## Author contributions

AV and JK wrote the final version. All authors reviewed and edited the manuscript and approved the final version of the manuscript.
